# Bidirectional Association Between Psoriasis and Nonalcoholic Fatty Liver Disease: Real-World Evidence From Two Longitudinal Cohort Studies

**DOI:** 10.3389/fimmu.2022.840106

**Published:** 2022-02-16

**Authors:** Shuo-Yan Gau, Kuang-Hua Huang, Chiu Hsiang Lee, Yu-Hsiang Kuan, Tung-Han Tsai, Chien-Ying Lee

**Affiliations:** ^1^ School of Medicine, Chung Shan Medical University, Taichung, Taiwan; ^2^ Department of Health Services Administration, China Medical University, Taichung, Taiwan; ^3^ School of Nursing, Chung Shan Medical University, Taichung, Taiwan; ^4^ Department of Nursing, Chung Shan Medical University Hospital, Taichung, Taiwan; ^5^ Department of Pharmacology, Chung Shan Medical University, Taichung, Taiwan; ^6^ Department of Pharmacy, Chung Shan Medical University Hospital, Taichung, Taiwan

**Keywords:** psoriasis, NHIRD, cohort study, non-alcoholic fatty liver disease (NAFLD), longitudinal study

## Abstract

**Background:**

Association between nonalcoholic fatty liver disease (NAFLD) and future psoriasis has not yet been confirmed, although the two diseases partially share a common pathogenesis pathway. Studies have revealed an association between psoriasis and subsequent NAFLD; however, these studies were limited to small sample sizes and a cross-sectional study design. Hence, the main objective of this population-based longitudinal cohort study was to evaluate the bidirectional association between psoriasis and NAFLD.

**Methods:**

Data were retrieved from Taiwan’s National Health Insurance Research Database. Patients with new-onset NAFLD and psoriasis were respectively enrolled in two cohorts. For each comparison cohort, propensity-score-matched controls with no record of NAFLD or psoriasis were selected. An adjusted hazard ratio (aHR) was applied to evaluate subsequent risks.

**Results:**

The risk of patients with new-onset NAFLD developing psoriasis was statistically significant, with an HR of 1.07 (95% CI, 1.01–1.14). For younger patients with NAFLD, the risk of developing psoriasis was 1.3-fold higher. The risk of patients with new-onset psoriasis developing NAFLD in the future was 1.28-fold higher than that of patients without psoriasis (95% CI, 1.21–1.35), and patients in younger psoriasis subgroups below the age of 40 years were at a higher risk than those in older subgroups, with an aHR of 1.55 (95% CI, 1.40–1.71).

**Conclusion:**

Evidence supports a bidirectional association between NAFLD and psoriasis, especially in patients below the age of 40 years. The correlation between the two diseases and the subsequent risk of disease development should be considered when caring for patients.

## Highlights

1. Nonalcoholic fatty liver disease is reported to have high prevalence in patients with psoriasis. The present study provides real-world evidence of the bidirectional association of increased incidence rate between the two diseases.
2. The correlation between psoriasis and nonalcoholic fatty liver disease and the subsequent risk of disease development should be considered when caring for patients.

## Introduction

Psoriasis is an immune-mediated skin disorder that presents with autoimmune characteristics (1). Obesity, diabetes, and stress are considered to be risk factors exacerbating psoriasis (2). The prevalence of psoriasis varies across regions. Approximately 2% of the US population has been reported to have psoriasis (3), whereas its prevalence has been estimated to be 0.235% in the Taiwanese population (4).

Nonalcoholic fatty liver disease (NAFLD) consists of different subgroups, including nonalcoholic fatty liver and nonalcoholic steatohepatitis (5), and is defined as a patient with low or no alcohol consumption presenting with >5% steatosis in hepatocytes (6). The prevalence of NAFLD has increased considerably in recent years, with a 33.90 prevalence rate reported in a recent meta-analysis (7). NAFLD increases mortality rates, with an overall mortality rate of more than 5 deaths per 1,000 person-years (7–9). An association between NAFLD and psoriasis has long been discussed. Given that the two diseases share some pathogenesis pathways, the prevalence of NAFLD has been widely reported to be high in patients with psoriasis (10, 11). Previous review studies have assumed that visceral obesity, insulin resistance, and elevated proinflammatory cytokines play critical roles in the mechanism of the association between the two diseases (12–15).

Available observational studies discussing the psoriasis–NAFLD association have mostly been limited to small sample sizes and a cross-sectional study design (14, 16–19). Moreover, regarding the inversed relationship, although a possible association has been assumed (12), real-world evidence regarding the risk of psoriasis in patients with NAFLD is lacking. Therefore, we conducted two longitudinal cohort studies to provide further evidence regarding this association.

## Material and Methods

### Data Source

Datasets for this retrospective population-based longitudinal cohort study were retrieved from Taiwan’s National Health Insurance Research Database (NHIRD). All medical claims in Taiwan are made through the National Health Insurance (NHI) program, which is a mandatory single-payer system. The NHI program has registered more than 99% of Taiwan’s 23 million citizens. The NHIRD contains claims data such as outpatient visits, hospitalizations, and admissions. Diagnosis and treatments are recorded using codes from the *International Classification of Disease, 9th revision, Clinical Modification* (*ICD-9-CM*) and *International Classification of Disease, 10th revision, Clinical Modification* (*ICD-10-CM*). The NHIRD has been widely used for epidemiological research (20, 21). To guarantee its validity and comprehensiveness, the NHI Bureau and Ministry of Health and Welfare in Taiwan administers the database. Informed consent is exempted when using NHIRD data because all personal information has been de-identified.

The present study was performed based on the NHIRD claim data between 2000 and 2018. To ensure at least five years of follow-up for each individual and data quality, we enrolled patients who had received a diagnosis of NAFLD and psoriasis between 2004 to 2013 and those who had not for the two cohorts in this study.

### Study Population, Exposure, and Outcomes

#### Study 1: NAFLD–Psoriasis Cohort

##### Patient Selection

**Figure 1:**
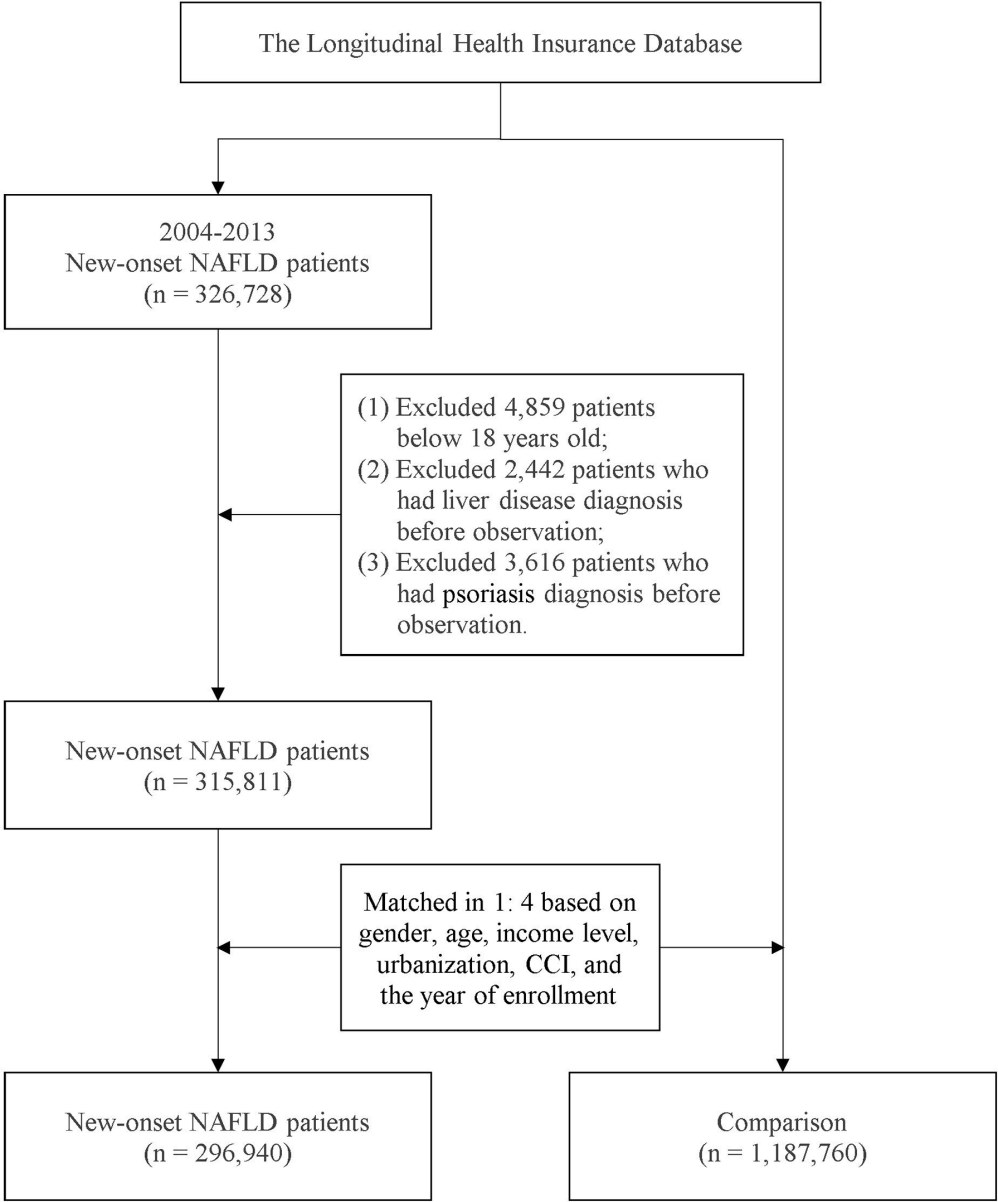
Patient selection for the NAFLD–psoriasis cohort.

Patients with a record of more than two outpatient visits or one inpatient visit for NAFLD were included in the NAFLD cohort. This definition has been applied in previous population-based studies in Taiwan (4, 22). Patients below 18 years old at the index date and those who had died or withdrawn from the NHI program before the study endpoint were excluded. Additionally, to address potential confounding bias, individuals with a history of liver diseases and psoriasis before the index date were not included in the study design. Based on propensity score matching (PSM), each individual in the NAFLD cohort was matched with four controls without NAFLD. Through a logistic regression model, matching variables including age, sex, year of enrollment, and comorbidities were calculated to determine the propensity score. The index date of the NAFLD cohort was set as the date of the first NAFLD diagnosis, whereas a random date between 2004 and 2013 was set as the index date for the patients without NAFLD. **Figure 1** provides detailed information on the recruitment of the study participants. All the individuals were followed up from the index date to the occurrence of outcome events, death, withdrawal from the NHI program, or the study endpoint on December 31, 2018.

##### Outcome Measures and Covariates

The occurrence of psoriasis was set as the endpoint of Study 1. To enhance the validity, only those with more than three outpatient visits based on the diagnosis of the dermatology department were considered to have psoriasis. This definition has been validated in previous studies using NHIRD (23, 24). To address potential confounders, variables including age, sex, income level, urbanization status, Charlson comorbidity index (CCI) scores, and comorbidities were considered. Comorbidities included hypertension, diabetes milieus, hyperlipidemia, myocardial infarction, coronary artery disease, obesity, alcoholism, major depressive disorder, rheumatoid arthritis, ankylosing spondylitis, and inflammatory bowel disease.

#### Study 2: Psoriasis–NAFLD Cohort

##### Patient Selection

**Figure 2:**
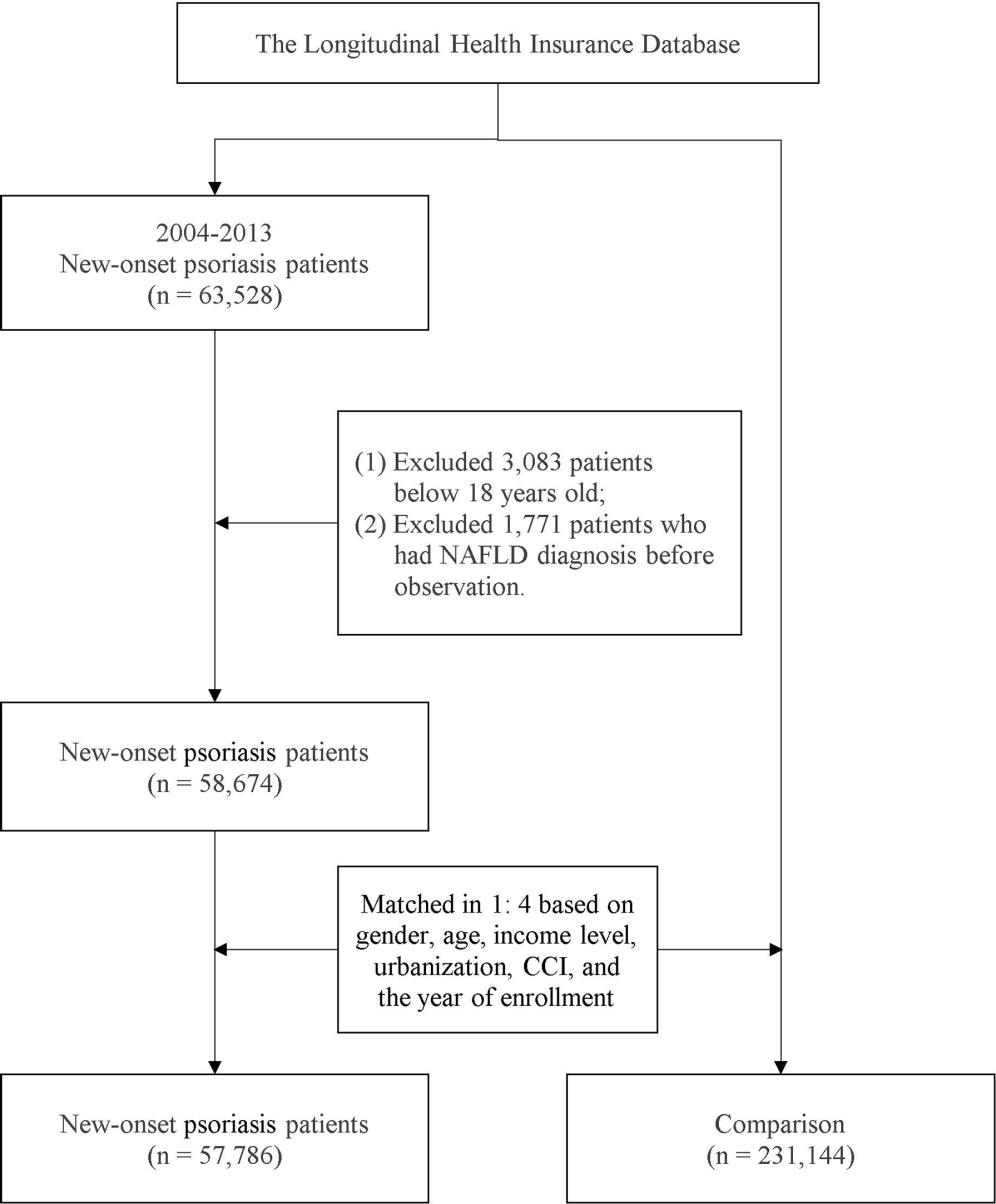
Patient selection for the psoriasis–NAFLD cohort.

To evaluate whether a bidirectional association between NAFLD and psoriasis exists, we designed a psoriasis cohort parallel to the NAFLD cohort. In Study 2, eligible patients with first-time psoriasis were recruited from the NHIRD. The study design was the same as that for Study 1; PSM was performed to recruit individuals without psoriasis as controls, and potential confounders were considered in the calculation of the propensity score. A detailed selection flowchart is presented in **Figure 2**. The index date for individuals in the psoriasis group was set as that of the first-time diagnosis of psoriasis; for the non-psoriasis group, a random date between 2004 and 2013 was set as the index date. All individuals in this analysis were followed up from the index date to the of outcome event occurrence, death, withdrawal from the NHI program, or the endpoint on December 31, 2018.

##### Outcome Measures and Covariates

The occurrence of NAFLD was set as the endpoint of Study 2. Variables including age, sex, income level, urbanization status, CCI scores, and related comorbidities, including hypertension, diabetes milieus, hyperlipidemia, myocardial infarction, coronary artery disease, chronic kidney disease, obesity, alcoholism, major depressive disorder, rheumatoid arthritis, ankylosing spondylitis, and inflammatory bowel disease were considered.

### Subgroup Analysis

To evaluate the difference between different age and the bidirectional NAFLD-psoriasis association, in subgroup analysis, people in Study 1 and Study 2 were stratified based on different age on the index date. All patients were categorized into three age subgroups—people below 40 years old, people between 41 to 64 years old and people above 65 years old. Respective aHR of each age subgroups comparing with the controls would be calculated.

### Statistical Analysis

A p value of <.05 was considered a significant difference. In the current population-based study, standardized mean difference (SMD) were also applied to present the difference in baseline characteristics to address the potential bias. With an SMD value <.1, the difference was negligible. Using the Cox model, the hazard ratio (HR) and 95% confidence interval (CI) were estimated and adjusted. SAS software version 9.4 (SAS Institute, Cary, NC, USA) was used to perform the statistical analysis.

### Statement of Ethics

This study was approved by the Central Regional Research Ethics Committee of China Medical University, Taiwan, with the IRB permit number CRREC-109-011. The current study conformed to the principles of the Declaration of Helsinki. All personal information was censored to protect the privacy of study participants. Because NHIRD data are de-identified, patient consent was exempted.

## Results

### Study 1. Baseline Characteristics of the Participants in the NAFLD Cohort

In total, 1,484,700 individuals [including 1,187,760 patients without NAFLD (non-NAFLD cohort) and 296,940 with NAFLD (NAFLD cohort)] were included in this study. In the NAFLD cohort, the proportion of female to male patients was approximately 0.76. The mean age of participants in the NAFLD cohort was 50.4 years old. Patients with NAFLD were more likely to have comorbidities, including hypertension, hyperlipidemia, and obesity. After PSM, the difference in variables, including age, sex, and comorbidity status, between the NAFLD and non-NAFLD cohorts was negligible, as reported in **Supplementary Table 1**.

### Study 1. Risk of Patients With NAFLD Developing Psoriasis

The confounders were considered and the risk of psoriasis in both cohorts was evaluated using a multiple Cox proportional hazard regression analysis, as reported in **Supplementary Table 2** and **Table 1**. The incidence rates of psoriasis in the NAFLD and non-NAFLD cohorts were 0.41 and 0.38 per 1,000 person-years, respectively. After adjustment for related confounders, compared with the non-NAFLD cohort, the risk of patients with new-onset NAFLD developing psoriasis was significant, with an HR of **1.07** (95% CI, 1.01–1.14). The results of the multivariate Cox model revealed that comorbidities including hypertension, hyperlipidemia, and rheumatoid arthritis were significant risk factors for psoriasis. Moreover, higher CCI scores and the male sex were associated with a greater risk of psoriasis, whereas higher income levels were negatively associated with psoriasis occurrence.

**Table 1:** Multiple Cox proportional hazard regression for estimation of hazard ratios on psoriasis.

Variables	HR	95% CI	p-value
Patients			
Comparison (ref.)	1		
**NAFLD**	**1.07**	**1.01 - 1.14**	**0.046**
Gender			
Female (ref.)	1		
** Male**	1.73	1.63 - 1.83	<0.001
Income level			
Low income (≤21,000) (ref.)	1		
Middle income (21,001-33,000)	0.89	0.83 - 0.95	0.001
High income (≥33,001)	0.85	0.80 - 0.91	<0.001
Urbanization			
Level 1 (ref.)	1		
Level 2	1.01	0.95 - 1.08	0.790
Level 3	0.92	0.85 - 1.00	0.043
Level 4	0.85	0.77 - 0.93	0.001
Level 5	0.94	0.74 - 1.20	0.612
Level 6	0.84	0.70 - 1.00	0.049
Level 7	1.02	0.87 - 1.19	0.851
CCI score			
0 (ref.)	1		
1	1.31	1.21 - 1.41	<0.001
2	1.52	1.40 - 1.65	<0.001
≥3	1.40	1.30 - 1.52	<0.001
**Hypertension**			
No (ref.)	1		
Yes	**1.19**	1.12 - 1.27	<0.001
Diabetes			
No (ref.)	1		
Yes	1.01	0.94 - 1.09	0.783
**Hyperlipidaemia**			
No (ref.)	1		
Yes	**1.21**	1.12 - 1.29	<0.001
Myocardial infarction			
No (ref.)	1		
Yes	1.22	0.86 - 1.72	0.271
Coronary artery disease			
No (ref.)	1		
Yes	1.15	1.06 - 1.25	0.001
Chronic kidney disease			
No (ref.)	1		
Yes	0.85	0.68 - 1.06	0.153
Obesity			
No (ref.)	1		
Yes	1.17	0.86 - 1.59	0.310
Alcoholism			
No (ref.)	1		
Yes	0.82	0.50 - 1.34	0.420
Major depressive disorder			
No (ref.)	1		
Yes	1.31	1.05 - 1.62	0.017
**Rheumatoid arthritis**			
No (ref.)	1		
Yes	**2.12**	1.76 - 2.54	<0.001
Ankylosing spondylitis			
No (ref.)	1		
Yes	1.03	0.73 - 1.44	0.888
Inflammatory bowel disease			
No (ref.)	1		
Yes	0.71	0.27 - 1.90	0.498

The bolding value refers to statistically significant values.

### Study 1. Stratification Analysis of Age at the Index Date in the NAFLD Cohort

The subgroup analysis reported that for younger patients with NAFLD, the risk of developing psoriasis was 30% higher than that for patients without NAFLD (aHR = 1.30, 95% CI, 1.14–1.48). In other age subgroups, participants over 40 years old exhibited a nonsignificant association with psoriasis (**Table 2**).

**Table 2:** Stratified analysis for the adjusted hazard ratio (95% C.I.) stratified by age subgroups (**Study 1**).

Variables	NAFLD vs Comparison (ref.)		
	aHR^1^	95% CI	p-value
Age (year)			
** ≤40**	**1.30**	**1.14 - 1.48**	**<0.001**
41-64	1.03	0.95 - 1.12	0.484
≥65	0.93	0.80 - 1.07	0.307

^1^aHR, adjusted hazard ratio. Extraneous factors adjusted in the model contained all variables in **Table 3**. The bolding value refers to statistically significant values.

### Study 2. Baseline Characteristics of the Participants in the Psoriasis Cohort

In the psoriasis–NAFLD study, 288,930 individuals were recruited in total, with 231,144 patients enrolled in the non-psoriasis cohort and 57,785 in the psoriasis cohort. As reported in **Supplementary Table 3**, the mean age of the patients with psoriasis was 48.4 years old, and the ratio of women to men was approximately 0.57. More than half of the psoriasis patients had a relatively low income level. In the psoriasis cohort, patients were more likely to have comorbidities. After PSM, the difference between the baseline characteristics in the two cohorts was adjusted, revealing no difference (with all SMD values for each variable <.1).

### Study 2. Risk of Patients With Psoriasis Developing NAFLD

As reported in **Table 3**, the risk of patients with new-onset psoriasis developing NAFLD was 1.28-fold higher than that for patients without psoriasis (95% CI, 1.21–1.35). The incidence rate of NAFLD in patients with psoriasis was 3.07 per 1,000 person-years, whereas in the non-psoriasis cohort, the incidence of NAFLD was 2.37 per 1,000 person-years (**Supplementary Table 4**). The urbanization level did not exhibit a significant association with the occurrence of NAFLD. However, within the variables considered, comorbidities including hypertension, hyperlipidemia, obesity, and alcoholism were associated with an increased risk of an outcome event.

**Table 3:** Multiple Cox proportional hazard regression for estimation of hazard ratios on nonalcoholic fatty liver disease.

Variables	HR	95% CI	p-value
Patients			
Comparison (ref.)	1		
**Psoriasis**	**1.28**	**1.21 - 1.35**	**<0.001**
Gender			
Female (ref.)	1		
Male	1.23	1.17 - 1.30	<0.001
Income level			
Low income (≤21,000) (ref.)	1		
Middle income (21,001-33,000)	1.02	0.96 - 1.09	0.475
High income (≥33,001)	1.13	1.07 - 1.19	<0.001
Urbanization			
Level 1 (ref.)	1		
Level 2	0.97	0.92 - 1.03	0.313
Level 3	0.89	0.82 - 0.95	<0.001
Level 4	0.93	0.86 - 1.01	0.069
Level 5	1.01	0.84 - 1.21	0.949
Level 6	1.20	1.06 - 1.36	0.004
Level 7	0.90	0.78 - 1.04	0.152
CCI score			
0 (ref.)	1		
1	1.93	1.82 - 2.04	<0.001
2	2.31	2.15 - 2.48	<0.001
≥3	2.06	1.93 - 2.20	<0.001
**Hypertension**			
No (ref.)	1		
Yes	1.30	1.22 - 1.39	<0.001
Diabetes			
No (ref.)	1		
Yes	1.07	0.99 - 1.15	0.070
**Hyperlipidaemia**			
No (ref.)	1		
Yes	1.47	1.37 - 1.57	<0.001
Myocardial infarction			
No (ref.)	1		
Yes	0.49	0.28 - 0.85	0.011
Coronary artery disease			
No (ref.)	1		
Yes	1.05	0.97 - 1.14	0.233
Chronic kidney disease			
No (ref.)	1		
Yes	0.68	0.53 - 0.86	0.001
**Obesity**			
No (ref.)	1		
Yes	1.97	1.48 - 2.63	<0.001
**Alcoholism**			
No (ref.)	1		
Yes	1.72	1.18 - 2.50	0.005
Liver fibrosis and cirrhosis			
No (ref.)	1		
Yes	1.11	0.67 - 1.84	0.677

The bolding value refers to statistically significant values.

### Study 2. Stratification Analysis of Age at the Index Date in the Psoriasis Cohort

According to the results summarized in **Table 4**, as the age at the index date increases, the risk of patients with psoriasis developing NAFLD decreases. Older patients with psoriasis (over 65 years old) did not present a significantly higher risk of developing NAFLD. For patients in the younger psoriasis subgroups (under 40 and between 41 and 64 years old), the aHRs were 1.55 (95% CI, 1.40–1.71) and 1.30 (95% CI, 1.21–1.40), respectively.

**Table 4:** Stratified analysis for the adjusted hazard ratio (95% C.I.) stratified by age subgroups (**Study 2**).

Variables	Psoriasis vs Comparison (ref.)		
	aHR^1^	95% CI	p-value
Age (year)			
≤40	1.55	1.40 - 1.71	<0.001
41-64	1.30	1.21 - 1.40	<0.001
≥65	0.89	0.78 - 1.02	0.083

^1^aHR, adjusted hazard ratio. Extraneous factors adjusted in the model contained all variables in **Supplementary Table 4**.

## Discussion

The present longitudinal retrospective cohort study based on an Asian population evidenced a bidirectional association between NAFLD and psoriasis. Patients with NAFLD exhibited a 1.07-fold greater risk of developing psoriasis compared with the non-NAFLD cohort, whereas patients with psoriasis had a 1.28-fold greater risk of developing NAFLD.

The coexistence of NAFLD and psoriasis has been widely described and reported (19, 25, 26). To date, although biological mechanisms regarding the association have not been clarified, hypotheses relate primarily to the common proinflammatory cytokines and abnormal visceral adipose tissue involved in the pathogenesis of the two diseases (12–15). As psoriasis is currently being considered a systemic disease (27), in patients with psoriasis, the stimulation of keratinocytes activates chronic inflammation (28, 29), leading to the infiltration of dendritic cells, T lymphocytes, macrophages, and neutrophils, which in turn triggers the secretion of proinflammatory interleukin (IL)-17 cytokines. Psoriasis and NAFLD are both affected by the proinflammatory T-helper (Th)17 axis, leading to a shared mechanism in pathogenesis. The IL-17 family (including IL17-A and IL17-E) has been reported to be involved in the development of NAFLD in an animal model (30). Given that innate immune signaling could mediate metabolic inflammation, IL-1 family cytokines, serving as regulatory function for Th17 cell differentiation, was also recently recognized to be involved in NAFLD-related inflammation (31, 32). In recent studies, associations between IL-17 mediated inflammation and metabolic diseases such as obesity and diabetes and cardiovascular diseases such as atherosclerosis has also been reported (33, 34). At systemic level, IL-17 downregulates the expression of endothelial vascular cell adhesion molecule-1, leading to evolution of endothelial damage, which was crucial in the pathogenesis of atherosclerosis (33, 35, 36). Since Th17 lineage regulates metabolism and adipogenesis, proinflammatory cytokines could influence the onset of NAFLD (13). The major role of Th-17 lineage in liver were thought to be highly involved in the progression from NAFLD to the severe subtype of Nonalcoholic Steatohepatitis (NASH) (37, 38). Additionally, abnormalities in visceral adipose tissue are considered to be involved in the pathogenesis of both diseases (14). Due to expanded and inflamed abnormal adipocytes, increased adipocytokines such as TNF-α (39) and leptin (40, 41) and decreased adiponectin secretion (14, 42) could contribute to apoptosis and necrosis in hepatocytes, progressing to liver disease.

NAFLD was regarded as a multi-systemic disease, as a term “Metabolic-associated Fatty Liver Disease” has been suggested to be clinically applied (43). With multiple systems involved, related factors including obesity, metabolic status, saturated fatty acid (SFA) and inflamed visceral adipose tissue could potentially be involved in psoriatic onset. Obesity and metabolic syndrome were also reported to elevate the risk of psoriasis (44, 45), implicating that obesity could serve as a potential confounder in evaluating the association between NAFLD and psoriasis. However, after adjusting obesity as one of the covariates, the NAFLD-psoriasis association remained statistically significant in our study. Previously, Kunz et al. raised that saturated free fatty acid could both directly and indirectly arouse the activation and amplification of proinflammatory cytokines through binding to cytoplasmic epidermal fatty acid binding proteins and activating toll-like receptors (44). Moreover, studies have also hypothesized that an increase in the release of free fatty acid (FFA) from abnormal visceral adipose tissue could affect the development of psoriasis or influence the severity of skin lesions (12, 15). In the light of these hypotheses, the observed NAFLD-psoriasis association presented in the current study could be partially explained. However, the actual mechanism and interaction between SFA, FFA, visceral adipose tissue and psoriasis onset should be clarified in future studies. To the best of our knowledge, the present study is the first real-world study evaluating the risk of future psoriasis in patients with NAFLD. Compared with previous studies, which focused on the prevalence of NAFLD in patients with psoriasis, this study reported the bidirectional relationship and increased incidences between the two diseases. The reported bidirectional association could serve as preliminary evidence for the hypotheses stated in previous reviews, indicating that proinflammatory cytokines and visceral obesity could also play a role in the inverse relationship between the two diseases.

In Study 1, a stratification analysis identified a difference in psoriasis risk between age subgroups, with patients under 40 years old with NAFLD being more likely to develop psoriasis. This trend could be attributed to the stronger function of the immune system in the younger generation. The term “early-onset psoriasis (EOP)” was defined as incidence of psoriasis before the age of 40 (46); in this study, EOP appeared to be dominant in the youngest subgroup. Evidence from immunocytochemical investigations has suggested that compared with those with late-onset psoriasis, patients with EOP have greater lymphocytic infiltration (18). Given that proinflammatory cytokines could serve as a crucial mediator in the development of psoriasis in patients with NAFLD (12–14), a more effective immune system and enhanced immune reactions in the younger age subgroups could serve as a possible explanation for the increased risk of EOP in the younger generation.

Previous studies discussing the association between psoriasis and the risk of future NAFLD were primarily limited to a smaller sample size or a cross-sectional study design (16, 19, 47, 48). The association could be different in Western and Asian populations because of the differences in lifestyle and diet (13). Although a previous meta-analysis reported a slightly different odds ratio (OR) between different ethnic groups (OR in Western populations = 2.16, 95% CI, 1.19–3.93; OR in Asian populations = 2.06, 95% CI, 1.77–2.40) (10), the evidence is limited because only two studies have considered Asian populations from Taiwan and India, whereas the number of studies on Western populations is double that (4, 48). In a previous Taiwanese population-based study, Tsai et al. reported that patients with psoriasis had a high prevalence of fatty liver, with the adjusted relative risk of 2.27 (95% CI, 1.90–2.71) (4). However, that study was limited to a shorter follow-up time and potential confounding biases caused by a limited data set and unadjusted variables. Moreover, the data set was based on data from 1999 to 2008, which is relatively obsolete and unrepresentative of recent trends. Using an up-to-date population-based database including claims data from 2000 to 2018, the present study evaluated the Asian population using long-term follow-up. Based on the reported 1.28-fold aHR in our study, the association in previous studies on Asian populations could be overestimated because of residual confounders and a limited sample size.

Age difference could be an issue in evaluating the psoriasis–NAFLD relationship. According to a recent meta-analysis, age did not significantly serve as a risk factor for NAFLD in patients with psoriasis (11). However, previous studies did not provide sufficient information regarding age stratification. In 2014, a prospective cohort study conducted by van der Voort et al. reported a 46.2% prevalence of NAFLD in patients with psoriasis over 55 years old (17). However, because the study only enrolled an older population, the results could not be generalized to the whole population, and the difference between age subgroups could not be evaluated. In another study, Ogdie et al. used a robust English database to evaluate the association based on a retrospective cohort design, reporting a 2.23-fold risk of NAFLD occurrence (49). However, an age stratification analysis was unavailable. In the present study, an age stratification analysis has been conducted. Consistent with the trend reported in a recent meta-analysis, the stratification of age in our results demonstrated that increasing age did not increase the risk of NAFLD in patients with psoriasis. For patients with psoriasis under 40 years old, the risk of developing NAFLD was higher than that in other age subgroups, with an aHR of 1.55 (95% CI, 1.40–1.71).

The main strength of the present study is that it was based on a large sample size and longitudinal study design. Given that previous similar studies have been limited to a small sample size and cross-sectional study design (16, 17, 19, 47, 48), the current study provides further evidence for the observed association between the two diseases. By using recent data from a population-based database covering more than 23 million individuals and applying a longitudinal cohort design, the strength of the evidence has been enhanced.

However, the study has some limitations. First, unadjusted residual confounders could lead to potential biases. Because detailed information on lifestyle is unavailable in the NHIRD, we were not able to directly adjust critical variables such as body mass index or insulin resistance status, which could be potential confounders in the study. However, we adjusted for related variables, such as hyperlipidemia, obesity, and diabetes as comorbidities, to address this shortcoming. Second, as an administration code, *ICD-9-CM* and *ICD-10-CM* might not be precise enough to serve as definitions for NAFLD and psoriasis. Corey et al. (50) reported that as billing data and administrative code, ICD-9 codes might not be sufficient enough to define NAFLD comparing with clinical classification algorithms. To date, available validation Electronic Medical Record (EMR) study for NAFLD was only conducted mainly based on the population of white people, whereas validation study in Asian population was not available. In this case, whether or not ICD-9 and ICD-10 codes of NAFLD could show sufficient positive predict value in the Asian population-based database remains uncertain.

However, though limited by the available data in current datasets, in the present study, we did try our best to make the definition of NAFLD as convincible as possible. The code ICD-9-CM 571.8 has been utilized as NAFLD definition in previous studies (22, 51). Similarly, ICD-10-CM codes K75.0 (for NAFLD) and K76 (for NASH) were also applied by population-based studies (52, 53). Definitions relating to exposure, outcomes and covariates of diseases applied in this study were based on previous population-based observational studies (4, 22–24, 54–56). In addition to applying definition utilized by previous studies, to strengthen the validity of the definition, we also excluded people with less than one impatient or two outpatient records in our study. Third, because data on the severity of psoriasis and the comorbidities are not available in the NHIRD, we were not able to evaluate whether differences in severity would cause differences in the reported association. Readers should be cautious while interpreting the results of the current study. Interaction between psoriasis severity and NAFLD has long been discussed. Based on results from a cross-sectional study, van der Voort et al. stated that psoriasis severity might not influence the association between psoriasis and NAFLD (17). However, in other studies, prevalence of NAFLD has been reported to be significantly higher in people with higher severity of psoriasis (16). Similarly, evidences from case-control studies also indicated that severity of psoriasis could also potentially influence the prevalence of NAFLD, for comparing with mild psoriasis patients, people with severe psoriasis could have higher odds ratio of having NAFLD (10, 49). Moreover, coexistence of psoriasis and NAFLD could lead to elevated severity of NAFLD (57). Given that severity of psoriasis could play potential role between the two diseases, future studies should consider the mechanism influencing the pathogenesis and correlation between NAFLD and psoriasis and focus on the difference between different psoriasis severity subgroups and to what extent the severity difference would affect the bidirectional association. Nonetheless, though severity of psoriasis unavailable, by calculating and including the CCI scores as a variable, we addressed the confounding bias caused by comorbidity severity to a large extent.

## Conclusion

We reported a bidirectional association between NAFLD and psoriasis, especially in subgroups under 40 years of age. The correlation between the two diseases and the subsequent risk of disease development should be considered when caring for patients.

## Data Availability Statement

Data in this study was retrieved from Taiwan’s NHIRD. All data were administrated by the Taiwan National Health Insurance (NHI) Bureau. Because of the Personal Information Protection Act and related regulations, data sets are not publicly available. Requests to access the data can formally be submitted to the NHI Bureau (https://dep.mohw.gov.tw/DOS/cp-5119-59201-113.html).

## Ethics Statement

The studies involving human participants were reviewed and approved by Central Regional Research Ethics Committee of China Medical University (IRB permit number CRREC-109-011). Written informed consent for participation was not required for this study in accordance with the national legislation and the institutional requirements.

## Author Contributions

Study conception and design: S-YG, K-HH, T-HT, CL, and C-YL. Data acquisition: T-HT, K-HH, Y-HK, and C-YL. Data analysis and demonstration: S-YG, T-HT, and C-YL. Original draft preparation: S-YG, T-HT, K-HH, Y-HK, CL, and C-YL. All the authors involved in drafting or revising the article and approved of the submitted version.

## Funding

This research was funded by the Ministry of Science and Technology, Taiwan (MOST 110-2410-H-040-002), Chung Shan Medical University, Taiwan (CSMU-INT-109-07), and China Medical University, Taiwan (CMU110-MF-113).

## Conflict of Interest

The authors declare that the research was conducted in the absence of any commercial or financial relationships that could be construed as a potential conflict of interest.

## Publisher’s Note

All claims expressed in this article are solely those of the authors and do not necessarily represent those of their affiliated organizations, or those of the publisher, the editors and the reviewers. Any product that may be evaluated in this article, or claim that may be made by its manufacturer, is not guaranteed or endorsed by the publisher.

## Abbreviations

NAFLD, nonalcoholic fatty liver disease; aHR, adjusted hazard ratio; NHIRD, National Health Insurance Research Database; ICD-9-CM, International Classification of Disease, Ninth Revision, Clinical Modification; 95% CI, 95% confidence interval.
