# Supplementary material for: Bidirectional Association Between Psoriasis and Nonalcoholic Fatty Liver Disease: Real-World Evidence From Two Longitudinal Cohort Studies
Source: Front Immunol. 2022 Feb 16;13:840106. doi: 10.3389/fimmu.2022.840106 (PMC8889012; doi:10.3389/fimmu.2022.840106)
Supplement: Supplementary file 1 [file Table_1.docx]

| **Supplementary Table 1.**  Baseline characteristics of patients with nonalcoholic fatty liver disease after matching (**Study 1**). | | | | | | |
| --- | --- | --- | --- | --- | --- | --- |
| Variables | Comparison | | NAFLD | | p-value ^2^ | SMD ^3^ |
|  | N | % | N | % |  |  |
| Total | 1,187,760 | 100.00 | 296,940 | 100.00 |  |  |
| Gender ^1^ |  |  |  |  | <0.001 | 0.01 |
| Female | 508,930 | 42.85 | 128,471 | 43.26 |  |  |
| Male | 678,830 | 57.15 | 168,469 | 56.74 |  |  |
| Age (year) ^1^ (mean ± SD) | 50.26 ± 15.49 | | 50.24 ± 14.88 | | <0.001 | 0 |
| ≤40 | 298,484 | 25.13 | 75,451 | 25.41 |  |  |
| 41-64 | 685,928 | 57.75 | 169,106 | 56.95 |  |  |
| ≥65 | 203,348 | 17.12 | 52,383 | 17.64 |  |  |
| Income level ^1^ |  |  |  |  | 0.240 | 0.03 |
| Low income (≤21,000) | 531,269 | 44.73 | 133,257 | 44.88 |  |  |
| Middle income (21,001-33,000) | 288,476 | 24.29 | 72,120 | 24.29 |  |  |
| High income (≥33,001) | 368,015 | 30.98 | 91,563 | 30.84 |  |  |
| Urbanization ^1^ |  |  |  |  | <0.001 | 0 |
| Level 1 | 375,720 | 31.63 | 91,695 | 30.88 |  |  |
| Level 2 | 397,622 | 33.48 | 98,564 | 33.19 |  |  |
| Level 3 | 187,521 | 15.79 | 46,411 | 15.63 |  |  |
| Level 4 | 141,929 | 11.95 | 36,673 | 12.35 |  |  |
| Level 5 | 15,307 | 1.29 | 4,412 | 1.49 |  |  |
| Level 6 | 33,273 | 2.80 | 9,416 | 3.17 |  |  |
| Level 7 | 36,388 | 3.06 | 9,769 | 3.29 |  |  |
| CCI score ^1^ |  |  |  |  | 0.550 | 0 |
| 0 | 304,460 | 25.63 | 76,115 | 25.63 |  |  |
| 1 | 397,890 | 33.50 | 99,730 | 33.59 |  |  |
| 2 | 214,041 | 18.02 | 53,603 | 18.05 |  |  |
| ≥3 | 271,369 | 22.85 | 67,492 | 22.73 |  |  |
| Enrolled year ^1^ |  |  |  |  | 1.000 | 0 |
| 2004 | 106,068 | 8.93 | 26,517 | 8.93 |  |  |
| 2005 | 101,672 | 8.56 | 25,418 | 8.56 |  |  |
| 2006 | 108,004 | 9.09 | 27,001 | 9.09 |  |  |
| 2007 | 116,860 | 9.84 | 29,215 | 9.84 |  |  |
| 2008 | 117,468 | 9.89 | 29,367 | 9.89 |  |  |
| 2009 | 122,424 | 10.31 | 30,606 | 10.31 |  |  |
| 2010 | 125,336 | 10.55 | 31,334 | 10.55 |  |  |
| 2011 | 125,648 | 10.58 | 31,412 | 10.58 |  |  |
| 2012 | 131,772 | 11.09 | 32,943 | 11.09 |  |  |
| 2013 | 132,508 | 11.16 | 33,127 | 11.16 |  |  |
| Hypertension |  |  |  |  | <0.001 | 0.09 |
| No | 871,400 | 73.36 | 205,959 | 69.36 |  |  |
| Yes | 316,360 | 26.64 | 90,981 | 30.64 |  |  |
| Diabetes |  |  |  |  | <0.001 | -0.03 |
| No | 982,179 | 82.69 | 248,344 | 83.63 |  |  |
| Yes | 205,581 | 17.31 | 48,596 | 16.37 |  |  |
| Hyperlipidaemia |  |  |  |  | <0.001 | 0.14 |
| No | 978,563 | 82.39 | 227,989 | 76.78 |  |  |
| Yes | 209,197 | 17.61 | 68,951 | 23.22 |  |  |
| Myocardial infarction |  |  |  |  | <0.001 | -0.02 |
| No | 1,183,312 | 99.63 | 296,202 | 99.75 |  |  |
| Yes | 4,448 | 0.37 | 738 | 0.25 |  |  |
| Coronary artery disease |  |  |  |  | <0.001 | 0.06 |
| No | 1,070,318 | 90.11 | 261,770 | 88.16 |  |  |
| Yes | 117,442 | 9.89 | 35,170 | 11.84 |  |  |
| Chronic kidney disease |  |  |  |  | <0.001 | -0.06 |
| No | 1,167,431 | 98.29 | 293,998 | 99.01 |  |  |
| Yes | 20,329 | 1.71 | 2,942 | 0.99 |  |  |
| Obesity |  |  |  |  | <0.001 | 0.12 |
| No | 1,182,846 | 99.59 | 292,321 | 98.44 |  |  |
| Yes | 4,914 | 0.41 | 4,619 | 1.56 |  |  |
| Alcoholism |  |  |  |  | <0.001 | 0.06 |
| No | 1,185,258 | 99.79 | 295,101 | 99.38 |  |  |
| Yes | 2,502 | 0.21 | 1,839 | 0.62 |  |  |
| Major depressive disorder |  |  |  |  | <0.001 | 0.05 |
| No | 1,175,103 | 98.93 | 292,084 | 98.36 |  |  |
| Yes | 12,657 | 1.07 | 4,856 | 1.64 |  |  |
| Rheumatoid arthritis |  |  |  |  | <0.001 | -0.03 |
| No | 1,173,867 | 98.83 | 294,442 | 99.16 |  |  |
| Yes | 13,893 | 1.17 | 2,498 | 0.84 |  |  |
| Ankylosing spondylitis |  |  |  |  | 0.010 | 0.01 |
| No | 1,181,405 | 99.46 | 295,236 | 99.43 |  |  |
| Yes | 6,355 | 0.54 | 1,704 | 0.57 |  |  |
| Inflammatory bowel disease |  |  |  |  | <0.001 | 0.10 |
| No | 1,187,760 | 100.00 | 295,575 | 99.54 |  |  |
| Yes | 0 | 0.00 | 1,365 | 0.46 |  |  |
| ^1^ Matching variables |  |  |  |  |  |  |
| ^2^ Chi-square test |  |  |  |  |  |  |
| ^3^ Standardized mean difference |  |  |  |  |  |  |
