# Supplementary material for: Bidirectional Association Between Psoriasis and Nonalcoholic Fatty Liver Disease: Real-World Evidence From Two Longitudinal Cohort Studies
Source: Front Immunol. 2022 Feb 16;13:840106. doi: 10.3389/fimmu.2022.840106 (PMC8889012; doi:10.3389/fimmu.2022.840106)
Supplement: Supplementary file 2 [file Table_2.docx]

| **Supplementary Table 2**. Incident Psoriasis (**Study 1**). | | | | | | | |
| --- | --- | --- | --- | --- | --- | --- | --- |
| Variables | Incident Psoriasis | | | | | | |
|  | No | | Yes | | IR ^1^ | p-value ^2^ | SMD ^3^ |
|  | N | % | N | % |  |  |  |
| Total | 1,479,109 | 99.62 | 5,591 | 0.38 | 0.39 |  |  |
| Patients |  |  |  |  |  | 0.015 | 0.03 |
| Comparison | 1,183,360 | 99.63 | 4,400 | 0.37 | 0.38 |  |  |
| NAFLD | 295,749 | 99.60 | 1,191 | 0.40 | 0.41 |  |  |
| Gender |  |  |  |  |  | <0.001 | 0.23 |
| Female | 635,619 | 99.72 | 1,782 | 0.28 | 0.29 |  |  |
| Male | 843,490 | 99.55 | 3,809 | 0.45 | 0.46 |  |  |
| Income level |  |  |  |  |  | <0.001 | -0.09 |
| Low income (≤21,000) | 661,726 | 99.58 | 2,800 | 0.42 | 0.41 |  |  |
| Middle income (21,001-33,000) | 359,437 | 99.68 | 1,159 | 0.32 | 0.37 |  |  |
| High income (≥33,001) | 457,946 | 99.64 | 1,632 | 0.36 | 0.37 |  |  |
| Urbanization |  |  |  |  |  | <0.001 | -0.05 |
| Level 1 | 465,582 | 99.61 | 1,833 | 0.39 | 0.40 |  |  |
| Level 2 | 494,228 | 99.61 | 1,958 | 0.39 | 0.40 |  |  |
| Level 3 | 233,092 | 99.64 | 840 | 0.36 | 0.37 |  |  |
| Level 4 | 178,023 | 99.68 | 579 | 0.32 | 0.34 |  |  |
| Level 5 | 19,650 | 99.65 | 69 | 0.35 | 0.39 |  |  |
| Level 6 | 42,554 | 99.68 | 135 | 0.32 | 0.34 |  |  |
| Level 7 | 45,980 | 99.62 | 177 | 0.38 | 0.41 |  |  |
| CCI score |  |  |  |  |  | <0.001 | 0.13 |
| 0 | 379,472 | 99.71 | 1,103 | 0.29 | 0.30 |  |  |
| 1 | 495,703 | 99.61 | 1,917 | 0.39 | 0.39 |  |  |
| 2 | 266,446 | 99.55 | 1,198 | 0.45 | 0.45 |  |  |
| ≥3 | 337,488 | 99.59 | 1,373 | 0.41 | 0.42 |  |  |
| Hypertension |  |  |  |  |  | <0.001 | 0.12 |
| No | 1,073,614 | 99.65 | 3,745 | 0.35 | 0.35 |  |  |
| Yes | 405,495 | 99.55 | 1,846 | 0.45 | 0.48 |  |  |
| Diabetes |  |  |  |  |  | <0.001 | 0.06 |
| No | 1,226,010 | 99.63 | 4,513 | 0.37 | 0.37 |  |  |
| Yes | 253,099 | 99.58 | 1,078 | 0.42 | 0.45 |  |  |
| Hyperlipidaemia |  |  |  |  |  | <0.001 | 0.10 |
| No | 1,202,237 | 99.64 | 4,315 | 0.36 | 0.36 |  |  |
| Yes | 276,872 | 99.54 | 1,276 | 0.46 | 0.50 |  |  |
| Myocardial infarction |  |  |  |  |  | 0.002 | 0.04 |
| No | 1,473,956 | 99.62 | 5,558 | 0.38 | 0.39 |  |  |
| Yes | 5,153 | 99.36 | 33 | 0.64 | 0.69 |  |  |
| Coronary artery disease |  |  |  |  |  | <0.001 | 0.11 |
| No | 1,327,262 | 99.64 | 4,826 | 0.36 | 0.37 |  |  |
| Yes | 151,847 | 99.50 | 765 | 0.50 | 0.52 |  |  |
| Chronic kidney disease |  |  |  |  |  | 0.352 | -0.01 |
| No | 1,455,917 | 99.62 | 5,512 | 0.38 | 0.39 |  |  |
| Yes | 23,192 | 99.66 | 79 | 0.34 | 0.39 |  |  |
| Obesity |  |  |  |  |  | 0.306 | 0.01 |
| No | 1,469,618 | 99.62 | 5,549 | 0.38 | 0.39 |  |  |
| Yes | 9,491 | 99.56 | 42 | 0.44 | 0.46 |  |  |
| Alcoholism |  |  |  |  |  | 0.931 | 0 |
| No | 1,474,784 | 99.62 | 5,575 | 0.38 | 0.39 |  |  |
| Yes | 4,325 | 99.63 | 16 | 0.37 | 0.38 |  |  |
| Major depressive disorder |  |  |  |  |  | 0.034 | 0.03 |
| No | 1,461,679 | 99.62 | 5,508 | 0.38 | 0.39 |  |  |
| Yes | 17,430 | 99.53 | 83 | 0.47 | 0.49 |  |  |
| Rheumatoid arthritis |  |  |  |  |  | <0.001 | 0.08 |
| No | 1,462,836 | 99.63 | 5,473 | 0.37 | 0.38 |  |  |
| Yes | 16,273 | 99.28 | 118 | 0.72 | 0.74 |  |  |
| Ankylosing spondylitis |  |  |  |  |  | 0.505 | 0.01 |
| No | 1,471,084 | 99.62 | 5,557 | 0.38 | 0.39 |  |  |
| Yes | 8,025 | 99.58 | 34 | 0.42 | 0.44 |  |  |
| Inflammatory bowel disease |  |  |  |  |  | 0.614 | -0.01 |
| No | 1,477,748 | 99.62 | 5,587 | 0.38 | 0.39 |  |  |
| Yes | 1,361 | 99.71 | 4 | 0.29 | 0.30 |  |  |
| ^1^ The incidence rate of per 1,000 person-years | |  |  |  |  |  |  |
| ^2^ Chi-square test |  |  |  |  |  |  |  |
| ^3^ Standardized mean difference |  |  |  |  |  |  |  |
