# Supplementary material for: Bidirectional Association Between Psoriasis and Nonalcoholic Fatty Liver Disease: Real-World Evidence From Two Longitudinal Cohort Studies
Source: Front Immunol. 2022 Feb 16;13:840106. doi: 10.3389/fimmu.2022.840106 (PMC8889012; doi:10.3389/fimmu.2022.840106)
Supplement: Supplementary file 3 [file Table_3.docx]

| **Supplementary Table 3** .  Baseline characteristics of patients with psoriasis after matching (**Study 2**). | | | | | | |
| --- | --- | --- | --- | --- | --- | --- |
| Variables | Comparison | | Psoriasis | | p-value ^2^ | SMD ^3^ |
|  | N | % | N | % |  |  |
| Total | 231,144 | 100.00 | 57,786 | 100.00 |  |  |
| Gender ^1^ |  |  |  |  | 0.977 | 0 |
| Female | 83,693 | 36.21 | 20,927 | 36.21 |  |  |
| Male | 147,451 | 63.79 | 36,859 | 63.79 |  |  |
| Age (year) ^1^ (mean ± SD) | 48.12 ± 17.46 | | 48.40 ± 17.78 | | 1.000 | 0 |
| ≤40 | 81,162 | 35.11 | 20,290 | 35.11 |  |  |
| 41-64 | 101,596 | 43.95 | 25,399 | 43.95 |  |  |
| ≥65 | 48,386 | 20.93 | 12,097 | 20.93 |  |  |
| Income level ^1^ |  |  |  |  | 1.000 | 0 |
| Low income (≤21,000) | 120,030 | 51.93 | 30,007 | 51.93 |  |  |
| Middle income (21,001-33,000) | 53,856 | 23.30 | 13,464 | 23.30 |  |  |
| High income (≥33,001) | 57,258 | 24.77 | 14,315 | 24.77 |  |  |
| Urbanization ^1^ |  |  |  |  | 1.000 | 0 |
| Level 1 | 65,187 | 28.20 | 16,293 | 28.20 |  |  |
| Level 2 | 76,584 | 33.13 | 19,136 | 33.12 |  |  |
| Level 3 | 41,131 | 17.79 | 10,281 | 17.79 |  |  |
| Level 4 | 29,609 | 12.81 | 7,390 | 12.79 |  |  |
| Level 5 | 3,807 | 1.65 | 965 | 1.67 |  |  |
| Level 6 | 7,476 | 3.23 | 1,877 | 3.25 |  |  |
| Level 7 | 7,350 | 3.18 | 1,844 | 3.19 |  |  |
| CCI score ^1^ |  |  |  |  | 1.000 | 0 |
| 0 | 130,356 | 56.40 | 32,589 | 56.40 |  |  |
| 1 | 47,500 | 20.55 | 11,875 | 20.55 |  |  |
| 2 | 20,791 | 8.99 | 5,193 | 8.99 |  |  |
| ≥3 | 32,497 | 14.06 | 8,129 | 14.07 |  |  |
| Enrolled year ^1^ |  |  |  |  | 1.000 | 0 |
| 2004 | 25,480 | 11.02 | 6,370 | 11.02 |  |  |
| 2005 | 23,348 | 10.10 | 5,837 | 10.10 |  |  |
| 2006 | 23,204 | 10.04 | 5,801 | 10.04 |  |  |
| 2007 | 22,560 | 9.76 | 5,640 | 9.76 |  |  |
| 2008 | 21,200 | 9.17 | 5,300 | 9.17 |  |  |
| 2009 | 21,192 | 9.17 | 5,298 | 9.17 |  |  |
| 2010 | 22,056 | 9.54 | 5,514 | 9.54 |  |  |
| 2011 | 23,156 | 10.02 | 5,789 | 10.02 |  |  |
| 2012 | 24,312 | 10.52 | 6,078 | 10.52 |  |  |
| 2013 | 24,636 | 10.66 | 6,159 | 10.66 |  |  |
| Hypertension |  |  |  |  | <0.001 | 0.07 |
| No | 181,995 | 78.74 | 43,882 | 75.94 |  |  |
| Yes | 49,149 | 21.26 | 13,904 | 24.06 |  |  |
| Diabetes |  |  |  |  | <0.001 | 0.04 |
| No | 205,631 | 88.96 | 50,752 | 87.83 |  |  |
| Yes | 25,513 | 11.04 | 7,034 | 12.17 |  |  |
| Hyperlipidaemia |  |  |  |  | 0.006 | 0.01 |
| No | 204,194 | 88.34 | 50,809 | 87.93 |  |  |
| Yes | 26,950 | 11.66 | 6,977 | 12.07 |  |  |
| Myocardial infarction |  |  |  |  | 0.415 | 0 |
| No | 230,514 | 99.73 | 57,617 | 99.71 |  |  |
| Yes | 630 | 0.27 | 169 | 0.29 |  |  |
| Coronary artery disease |  |  |  |  | <0.001 | 0.03 |
| No | 212,424 | 91.90 | 52,600 | 91.03 |  |  |
| Yes | 18,720 | 8.10 | 5,186 | 8.97 |  |  |
| Chronic kidney disease |  |  |  |  | <0.001 | 0.06 |
| No | 228,606 | 98.90 | 56,766 | 98.23 |  |  |
| Yes | 2,538 | 1.10 | 1,020 | 1.77 |  |  |
| Obesity |  |  |  |  | <0.001 | 0.02 |
| No | 230,548 | 99.74 | 57,556 | 99.60 |  |  |
| Yes | 596 | 0.26 | 230 | 0.40 |  |  |
| Alcoholism |  |  |  |  | <0.001 | 0.03 |
| No | 230,763 | 99.84 | 57,608 | 99.69 |  |  |
| Yes | 381 | 0.16 | 178 | 0.31 |  |  |
| Liver fibrosis and cirrhosis |  |  |  |  | <0.001 | 0.05 |
| No | 230,895 | 99.89 | 57,599 | 99.68 |  |  |
| Yes | 249 | 0.11 | 187 | 0.32 |  |  |
| ^1^ Matching variables |  |  |  |  |  |  |
| ^2^ Chi-square test |  |  |  |  |  |  |
| ^3^ Standardized mean difference |  |  |  |  |  |  |
