# Supplementary material for: Bidirectional Association Between Psoriasis and Nonalcoholic Fatty Liver Disease: Real-World Evidence From Two Longitudinal Cohort Studies
Source: Front Immunol. 2022 Feb 16;13:840106. doi: 10.3389/fimmu.2022.840106 (PMC8889012; doi:10.3389/fimmu.2022.840106)
Supplement: Supplementary file 4 [file Table_4.docx]

| **Supplementary Table 4.** Incident nonalcoholic fatty liver disease (**Study 2**). | | | | | | | |
| --- | --- | --- | --- | --- | --- | --- | --- |
| Variables | Incident NAFLD | | | | | | |
|  | No | | Yes | | IR ^1^ | p-value ^2^ | SMD ^3^ |
|  | N | % | N | % |  |  |  |
| Total | 281,767 | 97.52 | 7,163 | 2.48 | 2.51 |  |  |
| Patients |  |  |  |  |  | <0.001 | 0.11 |
| Comparison | 225,728 | 97.66 | 5,416 | 2.34 | 2.37 |  |  |
| Psoriasis | 56,039 | 96.98 | 1,747 | 3.02 | 3.07 |  |  |
| Gender |  |  |  |  |  | <0.001 | 0.11 |
| Female | 102,401 | 97.88 | 2,219 | 2.12 | 2.15 |  |  |
| Male | 179,366 | 97.32 | 4,944 | 2.68 | 2.71 |  |  |
| Income level |  |  |  |  |  | <0.001 | 0.01 |
| Low income (≤21,000) | 146,257 | 97.48 | 3,780 | 2.52 | 2.41 |  |  |
| Middle income (21,001-33,000) | 65,858 | 97.83 | 1,462 | 2.17 | 2.45 |  |  |
| High income (≥33,001) | 69,652 | 97.32 | 1,921 | 2.68 | 2.80 |  |  |
| Urbanization |  |  |  |  |  | <0.001 | -0.03 |
| Level 1 | 79,370 | 97.41 | 2,110 | 2.59 | 2.63 |  |  |
| Level 2 | 93,320 | 97.49 | 2,400 | 2.51 | 2.54 |  |  |
| Level 3 | 50,268 | 97.77 | 1,144 | 2.23 | 2.26 |  |  |
| Level 4 | 36,109 | 97.59 | 890 | 2.41 | 2.41 |  |  |
| Level 5 | 4,651 | 97.46 | 121 | 2.54 | 2.56 |  |  |
| Level 6 | 9,065 | 96.92 | 288 | 3.08 | 3.11 |  |  |
| Level 7 | 8,984 | 97.72 | 210 | 2.28 | 2.33 |  |  |
| CCI score |  |  |  |  |  | <0.001 | 0.35 |
| 0 | 160,153 | 98.29 | 2,792 | 1.71 | 1.73 |  |  |
| 1 | 57,433 | 96.73 | 1,942 | 3.27 | 3.33 |  |  |
| 2 | 24,956 | 96.04 | 1,028 | 3.96 | 3.98 |  |  |
| ≥3 | 39,225 | 96.55 | 1,401 | 3.45 | 3.55 |  |  |
| Hypertension |  |  |  |  |  | <0.001 | 0.16 |
| No | 220,760 | 97.73 | 5,117 | 2.27 | 2.27 |  |  |
| Yes | 61,007 | 96.76 | 2,046 | 3.24 | 3.39 |  |  |
| Diabetes |  |  |  |  |  | <0.001 | 0.11 |
| No | 250,285 | 97.62 | 6,098 | 2.38 | 2.39 |  |  |
| Yes | 31,482 | 96.73 | 1,065 | 3.27 | 3.45 |  |  |
| Hyperlipidaemia |  |  |  |  |  | <0.001 | 0.18 |
| No | 249,123 | 97.69 | 5,880 | 2.31 | 2.31 |  |  |
| Yes | 32,644 | 96.22 | 1,283 | 3.78 | 4.08 |  |  |
| Myocardial infarction |  |  |  |  |  | 0.121 | -0.02 |
| No | 280,981 | 97.52 | 7,150 | 2.48 | 2.51 |  |  |
| Yes | 786 | 98.37 | 13 | 1.63 | 1.78 |  |  |
| Coronary artery disease |  |  |  |  |  | <0.001 | 0.08 |
| No | 258,615 | 97.58 | 6,409 | 2.42 | 2.45 |  |  |
| Yes | 23,152 | 96.85 | 754 | 3.15 | 3.19 |  |  |
| Chronic kidney disease |  |  |  |  |  | 0.028 | -0.03 |
| No | 278,277 | 97.51 | 7,095 | 2.49 | 2.51 |  |  |
| Yes | 3,490 | 98.09 | 68 | 1.91 | 2.14 |  |  |
| Obesity |  |  |  |  |  | <0.001 | 0.06 |
| No | 280,988 | 97.53 | 7,116 | 2.47 | 2.50 |  |  |
| Yes | 779 | 94.31 | 47 | 5.69 | 6.07 |  |  |
| Alcoholism |  |  |  |  |  | <0.001 | 0.04 |
| No | 281,237 | 97.53 | 7,134 | 2.47 | 2.50 |  |  |
| Yes | 530 | 94.81 | 29 | 5.19 | 5.39 |  |  |
| Liver fibrosis and cirrhosis |  |  |  |  |  | 0.110 | 0.02 |
| No | 281,347 | 97.52 | 7,147 | 2.48 | 2.51 |  |  |
| Yes | 420 | 96.33 | 16 | 3.67 | 4.01 |  |  |
| ^1^ The incidence rate of per 1,000 person-years | |  |  |  |  |  |  |
| ^2^ Chi-square test |  |  |  |  |  |  |  |
| ^3^ Standardized mean difference |  |  |  |  |  |  |  |
