# Supplementary material for: Bidirectional Association Between Psoriasis and Nonalcoholic Fatty Liver Disease: Real-World Evidence From Two Longitudinal Cohort Studies
Source: Front Immunol. 2022 Feb 16;13:840106. doi: 10.3389/fimmu.2022.840106 (PMC8889012; doi:10.3389/fimmu.2022.840106)
Supplement: Supplementary file 5 [file Table_5.docx]

**Supplementary Table 5. Utilized ICD-9/ICD-10 codes in the study**

| Diseases | ICD-9 | ICD10 |
| --- | --- | --- |
| Psoriasis | 696.0, 696.1 | L40 |
| Non-alcoholic fatty liver disease (NAFLD) | 571.8 | K76.0, K75.81 |
| Major depression disorder | 296.2, 296.3 | F33 |
| Rheumatoid arthritis | 714.0 | M05 |
| Ankylosing spondylitis | 720.0 | M45 |
| Hypertension | 401-405 | I10 |
| Diabetes | 250 | E10-E13 |
| Inflammatory bowel disease | 555, 556 | K50,K51 |
| Hyperlipidaemia | 272 | E78.2- E78.4 |
| Myocardial infarction | 410 | I21 |
| Coronary artery disease | 411-414 | I25.1 |
| Chronic kidney disease | 585 | N18 |
| Obesity | 278.0 | E66 |
| Alcoholism | 303.0, 303.9 | F10.20 |
| Liver fibrosis and cirrhosis | 571.2,571.6 | K74 |
